# 基于气相色谱-质谱法的广泛靶向代谢组学方法开发

**DOI:** 10.3724/SP.J.1123.2022.10003

**Published:** 2023-06-08

**Authors:** Yating WANG, Yang YANG, Xiulan SUN, Jian JI

**Affiliations:** 1.江南大学食品学院, 食品科学与技术国家重点实验室, 功能食品国家工程研究中心, 江南大学协同创新中心, 江苏 无锡 214122; 1. State Key Laboratory of Food Science and Technology, National Engineering Research Center for Functional Food, School of Food Science and Technology, Collaborative Innovation Center, Jiangnan University, Wuxi 214122, China; 2.新疆农业大学食品科学与药学学院, 新疆 乌鲁木齐 830000; 2. College of Food Science and Pharmacy, Xinjiang Agricultural University, Urumqi 830000, China

**Keywords:** 气相色谱-质谱法, 代谢组学, 广泛靶向, 代谢通路, 方法开发, gas chromatography-mass spectrometry (GC-MS), metabolomics, widely-targeted, metabolic pathway, method development

## Abstract

气相色谱-质谱法(GC-MS)的四极杆检测器具有扫描速率低、离子流失率高、浓度检测范围窄的特点,这些缺陷限制了该技术在代谢组学领域的广泛应用,因此亟需建立一种基于GC-MS的高覆盖率代谢组学分析方法。本文提出了一种基于GC-MS的广泛靶向代谢组学方法,广泛靶向代谢组学结合了靶向和非靶向的优点,可以实现对代谢物质的定性和半定量检测,该方法以The Fiehn library(FiehnLib)数据库中的代谢物质信息为基础,建立直链脂肪酸甲酯(FAMEs)的保留时间与FiehnLib数据库中的保留指数(RI)的关系,根据FiehnLib数据库中的保留指数计算数据库中代谢物质在具体实验条件下的保留时间;对比分析并确定保留时间的阈值为0.15 min,优化最佳扫描间隔为0.20 s;优化代谢物质的定量离子以避免出峰时间相近离子的干扰;最终构建了含有611种代谢物质的选择离子监测(SIM)方法表,这611种代谢物质覆盖了KEGG(Kyoto Encyclopedia of Genes and Genomes)中65%的代谢通路。与全扫描非靶向GC-MS方法相比,该广泛靶向GC-MS方法所检测的代谢物质数量增加20%~30%,信噪比提高15%~20%;稳定性试验结果表明,使用该方法分析样品时,84%的代谢物质保留时间的日内相对标准偏差(RSD)均小于2%,91%的代谢物质保留时间的日内RSD均小于3%;54%的代谢物质保留时间的日间RSD均小于2%,76%的代谢物质保留时间的日间RSD均小于3%;通过对常见生物样本的检测分析,证明该方法大大提升了被检测到的代谢物质的数量和信噪比,可扩展GC-MS在代谢组学中的应用范围。

## Abstract (en)

Gas chromatography-mass spectrometry (GC-MS) detectors are widely used detection instruments owing to their distinct advantages over other analytical techniques, including lower sample consumption, higher sensitivity, faster analysis speed, and simultaneous separation and analysis. Metabolomics is an important component of system physiology that concerns systematic studies of the metabolite spectrum in one or more biological systems, such as cells, tissues, organs, body fluids, and organisms. Unfortunately, conventional GC-MS detectors also feature low scan rates, high ion loss rates, and a narrow concentration detection range, which limit their applications in the field of metabolomics. Therefore, establishing a GC-MS-based metabolomic analysis method with wide coverage is of great importance. In this research, a widely-targeted metabolomics method based on GC-MS is proposed. This method combines the universality of untargeted metabolomics with the accuracy of targeted metabolomics to realize the qualitative and semi-quantitative detection of numerous metabolites. It does not require a self-built database and exhibits high sensitivity, good repeatability, and strong support for a wide range of metabolic substances. The proposed method was used to establish the relationship between the retention time of straight-chain fatty acid methyl esters (FAMEs) and their retention index (RI) in the FiehnLib database based on the metabolite information stored in this database. We obtained a linear relationship that could be described by the equation *y*=40878*x-*47530, *r*^2^=0.9999. We then calculated the retention times of metabolites in the FiehnLib database under the experimental conditions based on their RI. In this way, the effects of significant variations in peak retention times owing to differences in the chromatographic column, temperature, carrier gas flow rate, and so on can be avoided. The retention time of a substance fluctuates within a certain threshold because of variations in instrument performance, matrix interference, and other factors. As such, the retention time threshold of the substance must be determined. In this paper, the retention time threshold was set to 0.15 min to avoid instrument fluctuations. The optimal scan interval was optimized to 0.20 s (possible values=0.10, 0.15, 0.20, 0.25, and 0.30 s) because longer sampling periods can lead to spectral data loss and reductions in the resolution of adjacent chromatographic peaks, whereas shorter sampling periods can result in deterioration of the signal-to-noise ratio of the collected signals. The metabolite quantification ions were optimized to avoid the interference of quantification ion peak accumulation in the case of similar peak times, and a selected ion monitoring (SIM) method table was constructed for 611 metabolites, covering 65% of the metabolic pathways in the KEGG (Kyoto Encyclopedia of Genes and Genomes). The developed method covered 39 pathways, including glycolysis, the tricarboxylic acid cycle, purine metabolism, pyrimidine metabolism, amino acid metabolism, and biosynthesis. Compared with the full-scan untargeted GC-MS method, the widely-targeted GC-MS method demonstrated a 20%-30% increase in the number of metabolites detected, as well as a 15%-20% increase in signal-to-noise ratio. The results of stability tests showed that 84% of the intraday relative standard deviations (RSDs) of metabolite retention times were less than 2% and 91% of that were less than 3%; moreover, 54% of the interday RSDs of metabolite retention times were less than 2% and 76% of that were less than 3%. The detection and analysis results of common biological samples confirmed that the proposed method greatly improved the quantity and signal-to-noise ratio of the detected metabolites and is applicable to substances that are thermally stable, volatile, or volatile after derivation and have relative molecular masses lower than 600. Thus, the widely-targeted GC-MS method can expand the application scope of GC-MS in metabolomics.

代谢组学(metabolomics)是系统生理学的组成部分,它系统地研究了一个或多个生物系统(如细胞、组织、器官、体液或机体)中的代谢物谱^[1]^,并与其他组学数据结合,提高了对复杂细胞途径和生物学机制的理解^[2]^,它可以很好地应用于人类健康和疾病研究^[3,4]^、食品质量和作物生产研究^[5]^, 以及评估或管理污染对人类的影响^[6,7]^。其按研究目的不同可分为靶向代谢组学(targeted metabolomics)、非靶向代谢组学(untargeted metabolomics)和广泛靶向代谢组学(widely-targeted metabolomics)。

靶向代谢组学分析具有特异性强、检测灵敏度高和定量准确等特点^[8]^,不足之处在于物质的覆盖率有限。非靶向代谢组学用于已知和未知成分的整体比较,可以检测多种成分,具有较为广泛的物质覆盖率^[9,10]^,但由于缺乏标准品,可能会产生很多假阳性信号,缺乏对物质的绝对定性定量数据,同时该方法还存在光谱卷积、重复性差、检出限低等问题^[10⇓⇓-13]^。

广泛靶向代谢组学是一种整合了非靶向代谢组学“广泛性”和靶向代谢组学“准确性”优点的新型代谢组学检测技术,具有高通量、高灵敏度、广覆盖度、定性定量准确等特点,Li等^[14]^于2012年首次提出了广泛靶向代谢组学的概念。该方法首先通过全扫描(full scan)模式获取代谢物的质谱信息和离子对信息,然后采用选择离子监测(selected ion monitoring, SIM)模式或多反应监测(multiple reaction monitoring, MRM)模式对代谢物进行靶向采集。Yuan等^[15]^通过气相色谱-质谱(GC-MS)广泛靶向代谢组学方法对植物挥发物进行分析,建立了一个包含537个挥发物信号的数据库,并将该方法应用到番茄机械损伤评估中,发现与非靶向方法相比,广泛靶向方法灵敏度更高、物质覆盖率更高、重复性更好,该方法解决了卷积问题,采用MRM模式提高了灵敏度,并开发了回归模型来校正信号漂移,但需要自己构建数据库。Zhou等^[16]^应用GC-SIM-MS广泛靶向代谢组学方法对血浆进行代谢组学分析,选择3个特征离子进行检测,发现与非靶向方法相比,广泛靶向方法的相对标准偏差(RSD)值更小,皮尔逊(Pearson)相关性更好,信噪比提高了2~5倍,具有良好的重复性,满足代谢组学研究要求。

本研究开发了GC-MS广泛靶向代谢组学新方法,该方法无需自建数据库,在现有The Fiehn library (Fiehnlib)数据库的基础上进行调整;通过测定直链脂肪酸甲酯(fatty acid methyl esters, FAMEs)的保留时间(retention time, RT),将数据库中的保留指数(retention index, RI)换算成在具体实验条件下的保留时间,解决峰的保留时间会随着色谱柱类型、温度、载气流速等发生较大变化的问题;通过选择定量离子,避免出峰时间相近并且有相同特征离子的代谢物质互相干扰,适用于热稳定、易挥发或衍生化易挥发且相对分子质量低于600的物质,综合了full scan模式无偏向检测和SIM模式高响应值的优点,提高了灵敏度和数据质量,拓展了线性范围。

## 1 实验部分

### 1.1 仪器、试剂与材料

QP2010气相色谱-质谱联用仪和AOC-20i自动进样器(日本岛津);TL2010S高通量组织研磨仪(北京鼎昊源科技有限公司);AX224ZH电子天平(常州奥豪斯仪器有限公司);SB-5200D超声波清洗机(宁波新芝生物科技股份有限公司);VXMNFS涡旋仪(常州奥豪斯仪器有限公司);5424R离心机(上海艾本德中国有限公司);SCIENTZ-10N冷冻干燥机(宁波新芝生物科技股份有限公司);SCIENTZ-10LS真空浓缩仪(江苏秉宏生物科技有限公司);SHP-150恒温培养箱(上海森信实验仪器有限公司);Direct-Q 5UV超纯水仪(南京汉隆实验器材有限公司)。

乙腈、异丙醇、正己烷、氯仿(色谱纯,美国TEDIA公司);甲氧基胺盐酸盐(MeOx,分析纯,国药集团);双(三甲基硅基)三氟乙酰胺(BSTFA)+三甲基氯硅烷(TMCS)(99∶1,Supelco,分析纯)购自默克化工技术(上海)有限公司;实验用水为超纯水仪过滤后的水(18.2 MΩ·cm, 25 ℃)。

### 1.2 样品的制备

#### 1.2.1 样品的预处理

精确称量50 mg固体样品(如椰子叶、小鼠肝脏、鱼肉等)或50 μL液体样品(如发酵液等)于2 mL EP管中,准确加入0.5 mL乙腈-异丙醇-水(体积比为3∶3∶2)混合溶液;加入2~3个直径为2 mm的钢珠,放入高通量组织研磨仪中室温振荡超声5 min(液体样品不需此步骤);再加入0.5 mL乙腈-异丙醇-水(体积比为3∶3∶2)混合溶液,室温超声5 min; 14000 r/min离心2 min,取上清液500 μL加入到新的2 mL EP管中,然后使用真空浓缩仪浓缩至近干。

#### 1.2.2 MeOx+三甲基硅烷(TMS)衍生化

向浓缩近干的样品中加入80 μL 20 g/L的MeOx (溶剂为吡啶),涡旋振荡30 s, 于60 ℃反应60 min;再加入100 μL BSTFA+TMCS试剂, 于70 ℃条件下反应90 min, 14000 r/min离心3 min,取上清液120 μL加入到GC-MS检测瓶中的衬管里。

### 1.3 色谱-质谱条件

气相色谱 岛津RTx-5MS毛细管柱(30 m×0.25 mm×0.25 μm),载气流速1.53 mL/min。进样口温度240 ℃,程序升温条件:起始柱温50 ℃,维持1 min,以10 ℃/min升至320 ℃,维持5 min。进样分流比为10∶1,进样量1 μL。

质谱条件 电子轰击电离源(EI);电离能量70 eV;接口温度300 ℃;离子源温度300 ℃;溶剂延迟时间为4 min; SCAN扫描(即full scan模式)时,质量扫描范围*m/z* 85~500,扫描间隔为0.20 s; SIM扫描时,应用构建的包含611种代谢物质的SIM模式方法表(详见附表1,www.chrom-China.com),扫描间隔为0.20 s。

进样器条件 进样前、后溶剂(正己烷)冲洗次数均为4次,样品冲洗次数为2次;黏度补偿时间为0.20 s;柱塞速度、柱塞进样速度、进样器进样速度均为高速。

### 1.4 数据处理

#### 1.4.1 full scan数据处理

将full scan模式采集的质谱数据用岛津GC-MS工作站转换为mzXML格式,再使用Analysis Base File Converter将mzXML格式转换成abf格式。将其导入MS-DIAL4.20进行去卷积,最小峰高设置为3000,峰识别的保留指数容差为10000(Fiehn Index), EI图谱的相似度(dot product和reverse dot product)阈值设置为0.7,峰对齐的保留时间容差为0.1,其余参数为默认值。

#### 1.4.2 SIM数据处理

将SIM模式采集的质谱数据直接用岛津GC-MS工作站根据数据分析方法表(附表2,包括物质的名称、定量离子、保留时间、开始处理时间、结束处理时间)进行定量分析,对定量参数进行设定,保留时间阈值为0.15 min,最小峰高设置为3000,然后对峰面积定量。

## 2 结果与讨论

本文拟通过以FiehnLib数据库中的代谢物质信息为基础,建立FAMEs的保留时间与FiehnLib数据库中保留指数的关系,根据FiehnLib数据库中的保留指数计算数据库中代谢物质在本实验条件下的保留时间,通过与实际实验采集的代谢物保留时间进行对比分析,确定广泛靶向清单中代谢物的保留时间阈值。优化每个代谢物的定量离子,以确保两个保留时间接近的代谢物选择不同的定量离子,避免软件对定量离子进行积分时产生错误;并优化了该广泛靶向方法的最优质谱参数,分析了该方法中代谢物的代谢通路覆盖性和日内、日间精密度。

### 2.1 GC-MS广泛靶向代谢组学方法的构建与优化

#### 2.1.1 基于保留指数的保留时间的计算

将使用full scan模式分析测得的FAMEs保留时间(*x*, min)与FiehnLib数据库中C_8_~C_30_直链脂肪酸甲酯的保留指数(*y*)建立线性关系,得到线性方程*y*=40878 *x* -47530, *r*^2^=0.9999(见图1),并应用此线性关系计算出FiehnLib数据库中760个代谢物质的保留时间。

**图1:**
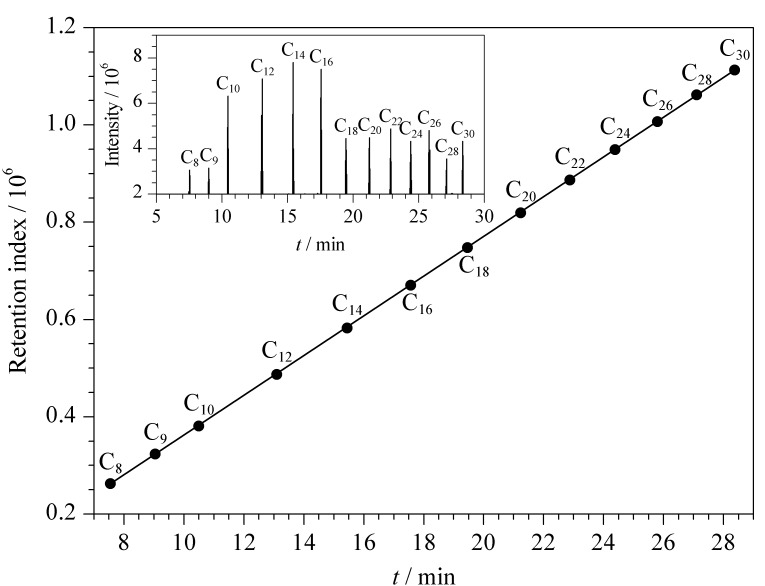
直链脂肪酸甲酯的保留指数和保留时间的拟合曲线(内 插图为FAMEs的提取离子流色谱图, *m/z* 87)

#### 2.1.2 保留时间阈值的确定

由于仪器的性能、基质的干扰等因素,物质的保留时间并不是确定的,而会在一定的阈值内波动,因此需要确定物质的保留时间阈值。以鱼肌肉样品为例,使用full scan模式进行分析,得到鱼肌肉的代谢物质种类及保留时间等数据。以理论计算的保留时间为横坐标,得到的保留时间与理论计算的保留时间差值为纵坐标,如图2所示,代谢物质的保留时间差在±0.15 min范围内,因此可以确定保留时间阈值为0.15 min。

**图2:**
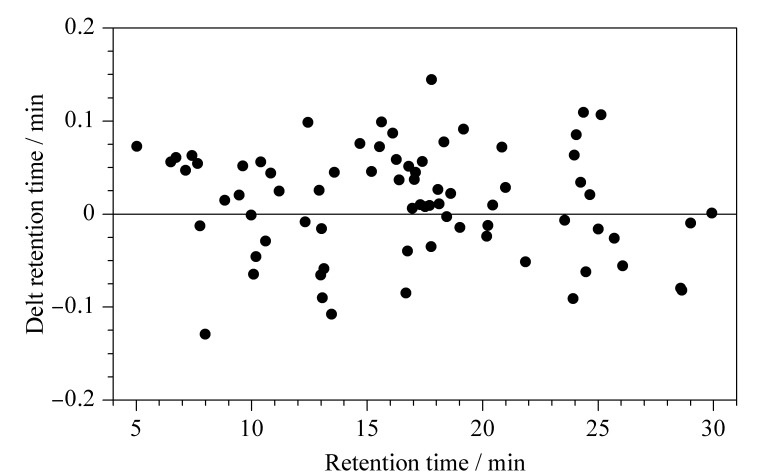
保留时间波动范围

#### 2.1.3 定量离子的选择

初步选择FiehnLib数据库中各代谢物质强度最大的特征离子作为定量离子,此时,由于各物质保留时间相近,且物质的保留时间在±0.15 min范围内波动,因此保留时间相近的物质若为同一定量离子时会互相干扰,以C_14_~C_16_直链脂肪酸甲酯保留时间范围内的代谢物质为例,不同代谢物质的特征离子的保留时间分布如图3a所示,存在代谢物质的定量离子保留时间重叠干扰的现象(如保留时间分别为15.87 min和15.88 min的高香草酸(homovanillic acid)和4-羟基扁桃酸(4-hydroxymandelic acid)特征离子均为267,在±0.15 min会相互干扰),这将影响进一步的定性分析。因此需要对存在保留时间重叠问题的定量离子进行重新选择,选择强度次之的定量离子,以确保在物质保留时间范围内的所有代谢物质没有重复的定量离子(如选择高香草酸的特征离子为强度次之的*m/z* 209, 4-羟基扁桃酸的特征离子为*m/z* 267),筛选后代谢物质特征离子的保留时间分布如图3b所示,此时解决了代谢物质定量离子保留时间重叠的问题。

**图3:**
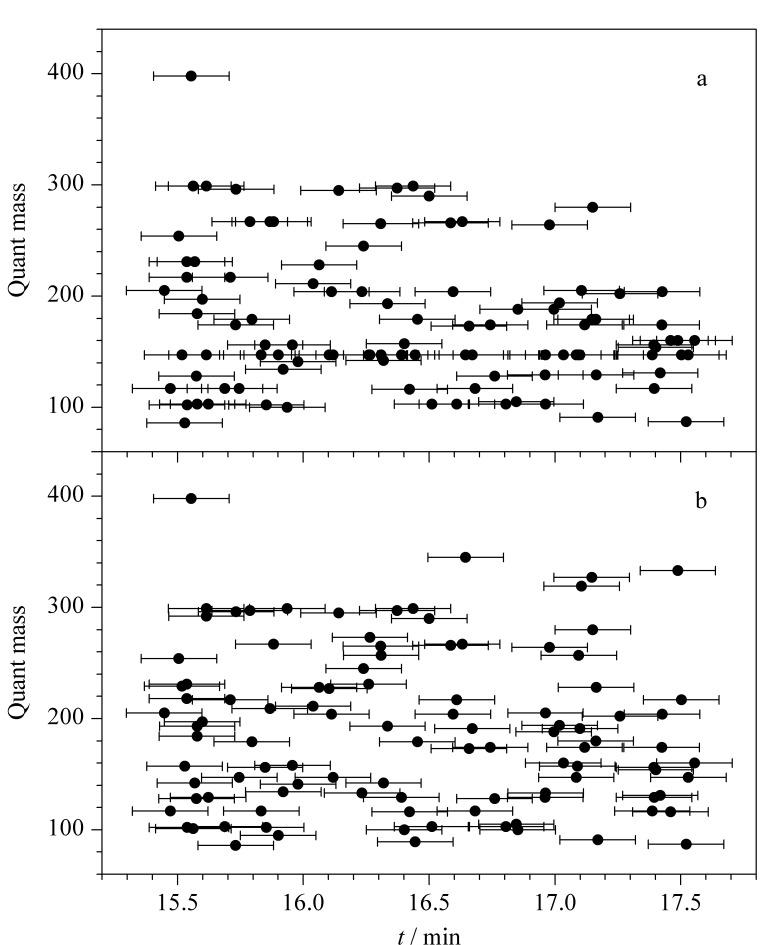
(a)筛选前与(b)筛选后代谢物质保留时间的分布

#### 2.1.4 方法表的构建与优化

数据库中的重复代谢物和非代谢物需要进行剔除,可以剔除57个非代谢物和92个重复代谢物,精简后数据库有611个物质。

由于GC-MS工作站每个通道中设置64个定量离子,因此需要设置多通道。本工作根据13个FAMEs的保留时间来进行通道设置,分别是C_8_之前组,C_8_~C_9_组,依次类推至C_28_~C_30_组,C_30_之后组,一共14组。由于物质的保留时间在±0.15 min范围内波动,因在保留时间节点前后的物质可能在前后两组均存在,所以需要对该类物质的通道设置做一下调整。

以C_8_脂肪酸甲酯保留时间节点为例,如表1所示,C_8_脂肪酸甲酯保留时间为7.580 min,(*S*)-2-羟基丁酸、草酸可能的保留时间范围既在C_8_脂肪酸甲酯前组中,也在C_8_~C_9_脂肪酸甲酯组中,因此这两个物质需要在两组都包含。同理,2-羟基-2-甲基丁酸、乳酰胺、肌氨酸的保留时间范围既在C_8_之前组中,也在C_8_~C_9_组中,所以这3个物质也需要在两组都包含。数据库中的所有代谢物质均按照该方法进行分组,建成含有611种代谢物质的SIM方法表(见附表1)。

**表1:** C_8_脂肪酸甲酯保留时间前后的物质的保留时间及范围

Substance	Retention time/min	Range of retention time/min
(S)-2-Hydroxybutyric acid	7.478	7.328-7.628
Oxalic acid	7.536	7.386-7.686
C_8_ fatty acid methyl ester	7.580	7.430-7.730
2-Hydroxy-2-methylbutanoic acid	7.641	7.491-7.791
Lactamide	7.663	7.513-7.813
Sarcosine	7.678	7.528-7.828

### 2.2 方法学考察结果

#### 2.2.1 灵敏度

对源于动物、植物、微生物的9种样品(羊肉、鱼肉、小鼠肝脏、小鼠上皮细胞(2个月和12个月)、植物黏液、椰子树叶、根系分泌物、发酵液)在full scan和SIM模式下分别进行分析,对两种方法测得的物质数量进行对比,如图4所示,SIM测得的物质数量明显增加。

**图4:**
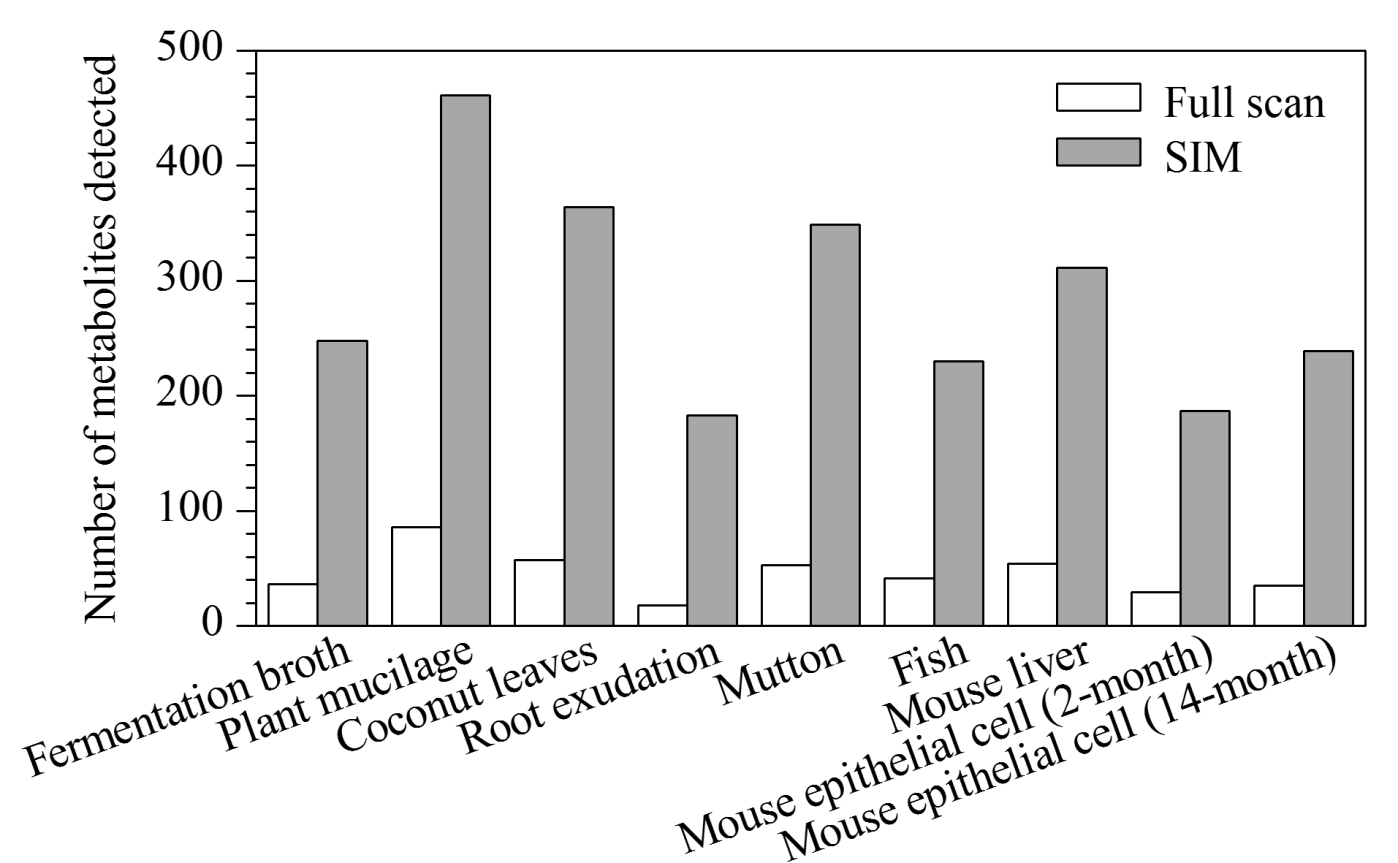
full scan和SIM方法测得的代谢物质数量对比

#### 2.2.2 扫描间隔的优化选择

扫描间隔即数据点的采样周期,采样周期设定值过大,会损失谱图细节,降低相邻色谱峰的分离度。采样周期设定值过小,会导致采集信号信噪比变差。本实验以藻类样品中的乳酸、甘油、棕榈酸、蔗糖为例(这4种代谢物质分别属于有机酸、糖醇、脂肪酸、糖类),选择了0.10、0.15、0.20、0.25、0.30 s 5个时间间隔进行分析,发现随着时间间隔的变大,即采样周期变长,信噪比会提高(图5a),但谱图的细节会变差(图5b~e),综合考虑谱图细节和信噪比两方面,扫描时间间隔选择为0.20 s。

**图5:**
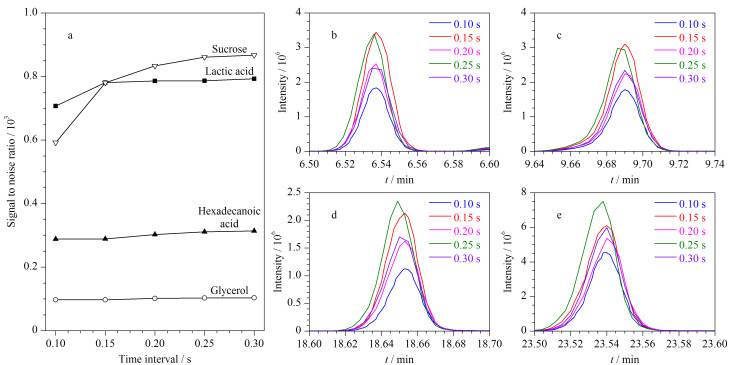
(a)信噪比随扫描间隔的变化以及(b)乳酸、(c)甘油、(d)棕榈酸、(e)蔗糖的总离子色谱图随时间间隔的变化

#### 2.2.3 覆盖通路

对筛选后含有611种代谢物质的数据库进行KEGG(Kyoto Encyclopedia of Genes and Genomes)通路覆盖分析,如图6所示,发现本方法覆盖了糖酵解、三羧酸循环、嘌呤代谢、嘧啶代谢、氨基酸代谢及生物合成等39种通路,其中代谢通路中代谢物的覆盖率在30%以上的通路数量占KEGG总代谢通路数量的65%。

**图6:**
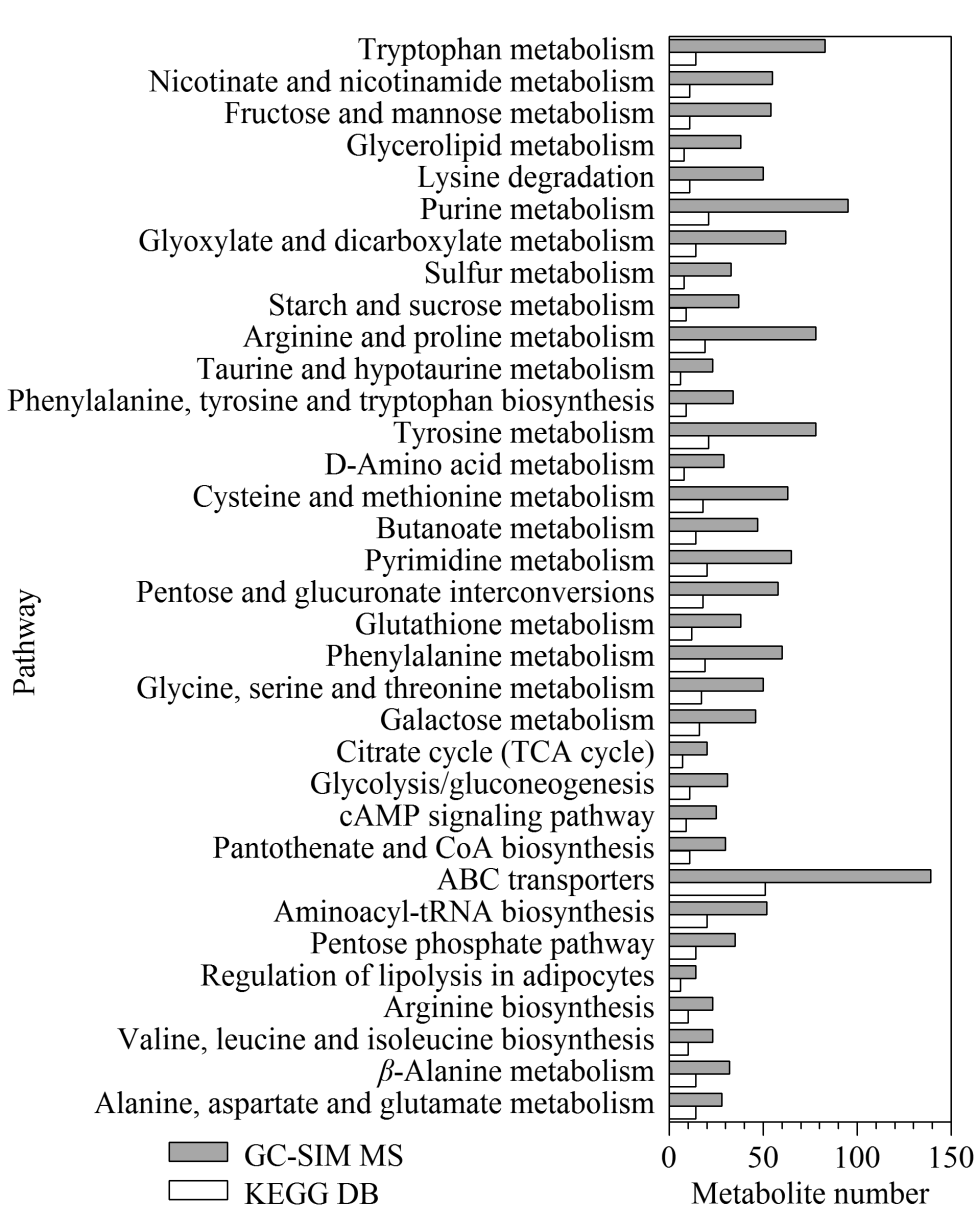
代谢物质的通路覆盖

### 2.3 数据可靠性分析

采用full scan模式分析鱼肌肉样品进行日内精密度的测试,在一天内进样6次,使用MS-DIAL 4.20对数据进行定性分析,得到鱼肌肉样品的代谢物质种类和保留时间,鱼肌肉样品代谢物质保留时间的日内RSD如图7a所示,可以看到84%的日内RSD均小于2%, 91%的日内RSD均小于3%;采用full scan模式分析藻类样品进行日间精密度的测试,连续分析3天,每天进样3次取平均值,使用MS-DIAL 4.20对数据进行定性分析,得到藻类样品的代谢物质种类和保留时间,藻类样品代谢物质保留时间的日间RSD分布如图7b所示,可以看到54%的日间RSD均小于2%, 76%的日间RSD均小于3%,仪器的日间和日内精密度均较高。

**图7:**
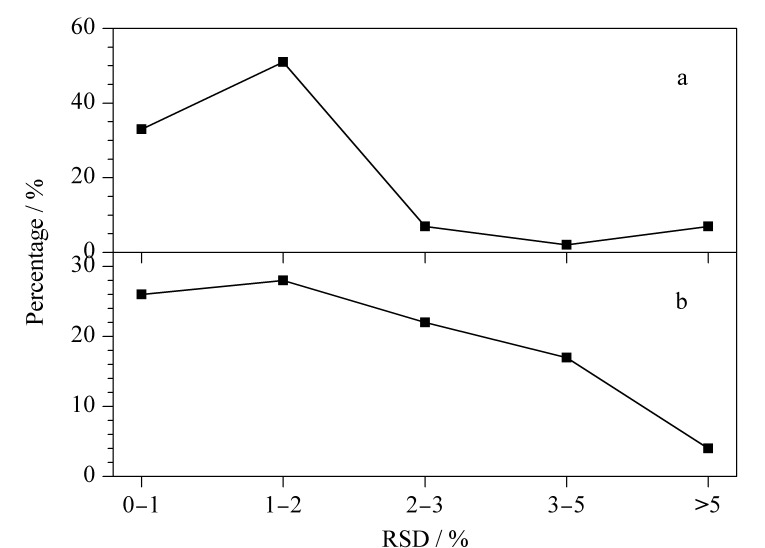
(a)日内和(b)日间RSD分布

## 3 结论

本实验开发了一种GC-MS广泛靶向代谢组学新方法,该方法无需自建数据库,通过测定FAMEs保留时间,与数据库中的保留指数建立线性关系,计算代谢物质的保留时间;确定保留时间波动阈值为0.15 min;人工选择定量离子,构建了含有611种代谢物质的SIM方法表;结果显示该广泛靶向方法较全扫描方法对样品中检测出来的代谢物数量有不同程度的提升。在最优扫描时间间隔为0.20 s的参数下,KEGG代谢通路覆盖率达65%,且该方法日间和日内精密度均较高。但该方法也存在一些不足有待进一步完善:(1)有些步骤需要人工筛选,工作量较为繁琐,比如定量离子的选择和SIM方法表的构建;此外使用GC-MS再解析软件对测量结果进行定性定量分析时,也会有假阳性的分析结果出现,这也需要人工筛选。(2)Fiehnlib数据库虽然代谢物质数量有600余种,但进行代谢通路分析时,每个通路的代谢物质种类会少于各通路涉及的所有代谢物质种类,这可能导致代谢通路结果的分析不够全面。
